# Banking on My Voice: Life with Motor Neurone Disease

**DOI:** 10.3390/healthcare13141770

**Published:** 2025-07-21

**Authors:** Ian Barry, Sarah El-Wahsh

**Affiliations:** 1 Australian Director of Film and TV, Sydney, NSW, Australia; 2 Person Living with MND, Sydney, NSW, Australia; 3 Speech Pathology, Aged Health Chronic Care & Rehabilitation, Concord Repatriation General Hospital, Hospital Road, Concord, Sydney, NSW 2139, Australia; 4 Speech Pathology, Faculty of Medicine and Health, The University of Sydney, Camperdown, Sydney, NSW 2050, Australia

**Keywords:** motor neurone disease (MND), progressive neurological disease, diagnosis, lived experience, multidisciplinary team, communication and identity, voice banking and synthetic voice, technology

## Abstract

This perspective paper presents a first-person account of life with motor neurone disease (MND). Through the lens of lived experience, it explores the complex and often prolonged diagnostic journey, shaped in part by the protective grip of denial. This paper then delves into the emotional impact of MND on the individual and their close relationships, capturing the strain on identity and family dynamics. It also highlights the vital role of the multidisciplinary team in providing support throughout the journey. A central focus of the paper is the personal journey of voice banking. It reflects on the restorative experience of reclaiming a pre-disease voice through tools such as ElevenLabs^TM^. This narrative underscores the critical importance of early intervention and timely access to voice banking, positioning voice not only as a tool for communication but also as a powerful anchor of identity, dignity, and agency. The paper concludes by highlighting key systemic gaps in MND care. It calls for earlier referral to speech pathology, earlier access to voice banking, access to psychological support from the time of diagnosis, and better integration between research and clinical care.

## 1. Introduction

Motor neurone disease (MND) is a progressive neurological disease characterised by the degeneration of motor neurons, leading to muscle weakness, loss of voluntary movement, and eventually respiratory failure [1,2]. The disease has a profound impact not only on physical function but also on communication and quality of life, affecting both the individuals diagnosed and their families [3]. Despite advances in understanding its pathophysiology, MND remains incurable, and timely diagnosis and supportive interventions are critical to managing its complex challenges [2].

This paper presents a lived experience perspective on MND, told through the personal story of Ian. Unlike traditional research papers or clinical case studies, this narrative offers unique insights grounded in Ian’s firsthand experiences, emotions, and reflections [4]. By sharing his journey, the article aims to deepen understanding of the personal and relational impact of MND, highlighting themes of resilience, hope, and the realities faced by some people living with MND and their loved ones. This perspective complements clinical knowledge and research by providing an authentic voice from within the MND community. Specifically, the article explores the diagnostic odyssey faced by patients with MND and places a central focus on the power of voice banking to preserve identity and agency in the face of speech changes. At the end of the article, Dr El-Wahsh, Ian’s speech pathologist, shares some of her insights on working with Ian and supporting people living with MND. These themes, explored through deeply personal accounts, can inform, challenge, and inspire change in clinical practice.

While this article is a personal reflection centred on Ian’s lived experience, relevant research literature has been integrated to support and contextualise insights. To maintain the authenticity of Ian’s narrative, citations have been added at points where statements align with established MND research, linking lived experience to the broader evidence base.

## 2. Life Before MND

Hanging from navy patrol boats, secured to buffeting helicopter doorways, descending into the steaming crater of Mount Pinatubo… there was no end to the crazy situations in which I would find myself! But this was back then. My life before MND was one of limitless physical joy, likely taken for granted and definitely underappreciated. I worked as a film and television director. It was a life that required me to travel anywhere, at any time, involving every imaginable mode of transport, and demanding a wide variety of operational tasks.

**Figure 1:**
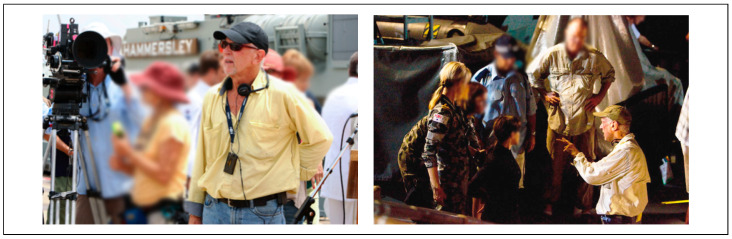
Life before MND: directing scenes on set for *Sea Patrol* (2007–2008).

Consequently, my directing role demanded constant agility—a nimbleness in precarious situations—and a high level of physical fitness and versatility. It also called for articulate and confident communication under pressure. Clear-headed people skills were essential, coupled with the ability to lead with authority and emotional intelligence. I often had to juggle posturing egos, manage actor insecurities, and effectively deploy crew resources. It was a profession that I loved every single day of my working life (see Figure 1). Then, in the final decade of my shooting career (2008–2018), everything began to change. My body slowly rebelled.

## 3. Initial Symptoms and My Diagnostic Journey

My first symptom appeared subtly in 2008: a slight slur in my speech—a blurred diction that concerned both my wife and daughter. Their observations prompted a visit to my GP, who referred me to a neurologist. Thus began my long and complex diagnostic journey [5,6,7]. After MRIs and electromyography, including needle tests in my tongue and neck, my first neurologist cautiously diagnosed me with Multiple System Atrophy (MSA), saying, “If you held a loaded gun to my head, I’d have to say MSA”. The words of this neurologist increasingly haunted my waking thoughts and my nightly dreams. The prospect of MSA had no positive outcomes and the deeply buried seed of suspicion he planted in me was slowly but surely taking root.

In the absence of a definitive diagnosis, I found a strange solace. While I could still function relatively normally, I chose to maintain the illusion of normality, masking minor fumbles and stumbles to keep living, working, and appearing unencumbered. Denial became my daily shield [8]. However, I came to understand something else about denial. While denial inevitably has its day of reckoning, along the way it can take a toll on those closest to you [8,9]. My wife, ever the realist, was carrying the emotional burden of my avoidance of reality. And so, I knew it was time for another trip into the MRI machine.

By late 2013, encouraged by my wife and another doctor, I returned to my original neurologist for a review assessment. This time, he revised his view: “You don’t have MSA”, the old sage said. “What do you mean I don’t have it?” I asked, eagerly pressing him for any vague glimmer of hope. “If you had MSA you would’ve come through that door in a wheelchair.” I felt momentarily relieved, released from one nightmare, only to realise there were more neurological monsters waiting in ambush just around the corner.

By 2015, beyond the physical deterioration in my legs, my speech had progressively worsened, though thankfully, it remained intelligible. My balance had assumed a steeper downhill trajectory. Before surgery for an arthritic knee, I sought clearance from a neurologist in the hospital. Within twenty minutes, this neurologist bluntly diagnosed me with MND and told me, “I wouldn’t bother with the knee operation”. His implication was imminent death. He had so devastated me that I drove straight to the studio where I was currently shooting and broke my television series contract, believing my career had ended. I have come to see how crucial it is for medical practitioners to choose their phraseology with care. One of the effects I have noticed of my MND is a disproportionate reaction to a given stimulus. These three letters, MND, would irrevocably change the rest of my life [10].

Soon after, shortness of breath emerged, and I was referred to a respiratory physician. He conducted extensive testing and, with some humour, gave me some surprising news: “I’m afraid to tell you all your results are normal”. His reassurance and measured response provided welcome relief after the starkness of previous consultations, spanning a full seven years. More importantly, he referred me to a neurologist in a specialist multidisciplinary MND Clinic for further investigations.

I passed through a litany of differential diagnosis, from MSA to ALS, SCA, and PLS. I gradually came to realise that MND can be a mysterious and elusive disease. My journey is not uncommon, and reflects the diagnostic complexity often involved in the early stages of MND, particularly in cases with slow progression [6,11].

Eventually, by late 2015, consensus emerged, and my neurologists settled on something closely resembling a final edict: Primary Lateral Sclerosis.

## 4. Symptom Progression and Emotional Impact

MND is, in essence, a process of anatomical demolition. Function declines as speech deteriorates and mobility weakens [1,12,13]. Eventually, communication becomes effortful and, for some, impossible [14]. For me, progression has been at the ‘slow’ end of the MND spectrum. For several years into my MND journey, I clung to denial as a way of resisting that change [8]. I convinced myself that denial was a kind of protective cloak. The opposite of denial is acknowledgement. Acknowledgement would give my condition credence. Credence would give it power, and by inverse logic, it would only make me weaker. So, I held on to denial for as long as I possibly could [8].

As my gait declined into a hesitant, unbalanced shuffle, I found myself inventing ways to compensate—adopting a rhythmic, swaying walk and continually experimenting with what I called “new tricks for old dogs”. I held back my deterioration with an ever-evolving repertoire of stunts. These improvisations offered temporary reprieve, but their effectiveness diminished over time. I began to recognise myself in cinematic figures like Lon Chaney’s *Frankenstein* or Ratso Rizzo from *Midnight Cowboy*—characters marked by physical limitation and fragility. In Schlesinger’s film, Ratso was dying from neglect, poor diet, and probably tuberculosis. He dreamt of escaping his affliction, running freely along a golden beach. Likewise, I found myself dreaming of physical freedom, longing for fluid speech, graceful movement, and effortless balance. I dreamt of speech with the diction of Richard Burton or Sir Laurence Olivier. But these aspirations remained just that—dreams.

## 5. My MND Secret

**Figure 2:**
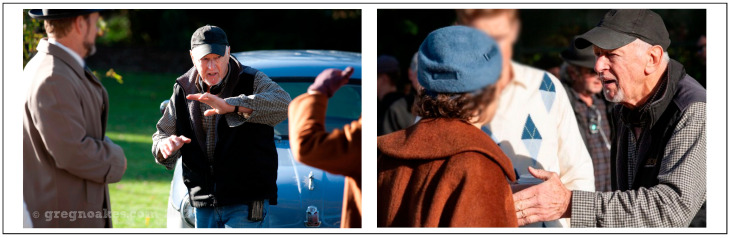
Life with MND: directing scenes on set for *The Doctor Blake Mysteries* (2012–2018).

By the beginning of 2016, the cracks in my façade of denial were visible to a widening circle of colleagues. In the final years of my on-set career, this self-deception became increasingly unsustainable. While shooting a popular murder mystery show in Melbourne (*The Doctor Blake Mysteries*; see Figure 2), I chose to stage a climactic scene on a steep staircase. Directing the action, up and down those stairs, became a hazardous exercise in balance. I knew people noticed my unsteadiness. It echoed earlier moments, like during *Sea Patrol,* when my slurred speech had prompted quiet speculation that I had a secret addiction to the bottle. The denial finally cracked when an actor, in a moment of levity, began mimicking my now slurred and nasalised diction.

At times, I felt the need to cover up and deflect questions with half-truths. While directing *Wild Boys* (2011), a colonial bushranger series, my line producer suspected I had had a stroke. I blamed my clearly faltering voice on arthritis in my neck, which x-rays had confirmed a couple of years previously. On *The Doctor Blake Mysteries*, I confided in a trusted producer friend and shared my diagnosis of slow-progressing MND. We made a pact that I would keep working for as long as possible—no matter what. I directed for three more fulfilling years, including *Blake* and *A Place to Call Home* (2015–2018). Then COVID-19 struck, and it gave me the perfect excuse to wholly hang up my viewfinder in 2020.

Slow progression does not spare you from eventual loss. Physical deterioration was accompanied by emotional strain and shifting relationships [9]. Carer fatigue often led to misunderstandings and emotional flare-ups [9]. Heightened emotional lability, common in MND, made laughter and tears come more easily [15]. To the non-afflicted, such emotional responses could seem exaggerated or false, adding a social weight to an already heavy burden.

In short, MND had taken the director’s seat in my life, leading me from denial and concealment to acknowledgement of my need for help.

## 6. My Support Landscape

Living with MND became a daily tightrope walk: each safe step, a small victory—each misstep, a potential catastrophe. Over the years, I have become well-acquainted with Emergency Departments across the state, from Sydney to Gosford to Lithgow, thanks to a string of falls and injuries often requiring stitches and urgent care.

Now, after more than a decade of managing both MND and age-related decline, the fatigue, both physical and emotional, has become profound. Depression creeps in as the trajectory becomes clearer and more irreversible [16]. You begin to question the point of persistence when every specialist you encounter confirms the same narrative—a slow descent: from walking stick to walking frame, from swallowing difficulties to feeding tubes, from independence to being pushed in a wheelchair just to experience a concert or festival. Holding onto optimism becomes increasingly difficult.

And yet, I remain deeply grateful for the multidisciplinary team who help me endure, some coming to my house, and others through hospital-based clinics. My dietitian supports me in maintaining weight and muscle mass. My speech pathologist helps me navigate the ongoing challenges of communication and swallowing. My occupational therapist adapted my home for safety and accessibility. My physiotherapist provides exercises to preserve what strength I still have. My podiatrist provided custom splints to support my feet and improve my stability, helping me walk more safely and confidently for as long as possible. The allied health support team felt like a cohesive well-oiled machine with frequent communication between all members [17].

From the medical side, my rehabilitation physician helps oversee and coordinate my overall function, ensuring that every part of my care plan aligns with my goals and changing needs. My respiratory physician monitors my lung function and provides crucial interventions to support breathing. As for neurology, I have had four different neurologists involved in my care. This was due to a combination of factors, including clinic closures, second opinions, and a move from private practice to the MND clinic. While each neurologist brought valuable expertise, they rarely communicated with my broader team. This reflects a broader issue in MND care: neurologists often operate in relative isolation, rather than as integrated members of a multidisciplinary team. I sometimes felt more like a lab rat than a patient, with neurologists placing a greater focus on research and cure rather than care. Overall, however, I have been fortunate to have had some consistent support over time from my care team—each of whom has played and continues to play a vital role, not only in supporting me but also in ensuring I feel heard and empowered by giving me real choice in my care [18]. Living with MND is, in many ways, a full-time job, but my care team’s collective expertise and encouragement make the load just that little bit lighter.

## 7. A Deposit in the Voice Bank

Part of my perfect storm of deterioration was the onset of bulbar symptoms, which affected both my speech and swallowing [14,19,20]. Bulbar changes can present differently in each person, but for most, the prospect of losing the ability to communicate is deeply confronting. Words are no longer reliable—pronunciation, volume, and conversational stamina carry a high degree of uncertainty. Will the words come out at all? If they do, they may be too soft, too loud, hoarse, high- or low-pitched, or carry the wrong emotional vale—merry when you want angry, or sad when you want celebration. There is no control over vocal output. You cannot predict the accuracy of your inflection or emotional colour, often leaving the recipient confused or uncomfortable. The reality is inescapable: living with bulbar symptoms is like navigating a linguistic obstacle course—never knowing whether your message will land or fall short. I long for the ability to express myself clearly again.

Since my MND, I can no longer just launch off the blocks into conversation with a sudden thought. Spontaneity is a thing of the past. I find I spend most gatherings waiting for a large enough gap in conversation to speak, but by the time I begin, someone else has taken the floor. I wait for the next gap, but by then the subject has changed. And so, I sit on the margins of group conversations, fatigued and robbed of participation. Group conversations become exhausting, not for lack of interest, but for the sheer effort required to participate.

I am living in a vocal minefield—and so I wish I had seen this coming and made a deposit in the voice bank.

## 8. My Voice Banking Experience

### 8.1. My Introduction

My first introduction to voice banking came through my speech pathologist (Sarah) in 2024, who had been supporting me to maximise the clarity of my increasingly impaired speech [20,21]. She gently and empathetically raised the possibility of preserving my vocal identity and capturing my voice before further deterioration rendered it unrecognisable. It was an idea I met with both curiosity and hesitation.

### 8.2. The First Software

The first software we used was basic and required me to record 50 pre-set phrases (and some additional custom phrases) to generate a synthetic voice for future text-to-speech use. The problem for me was that my synthetic voice reflected my already compromised speech: slow and blurred. I had already experienced nearly a decade of bulbar decline, so my recorded output embodied the very disability I was trying to outrun. Listening to the results only deepened my sense of loss. Each playback was a confronting reminder of how far my voice had fallen, leading me to question whether the whole voice banking process was worth continuing. It felt futile.

This was a limitation of that software: it captured the voice exactly as it was, reflecting the current state of speech, regardless of disease progression. Delaying the process can mean permanently banking a voice you no longer feel connected to. The emotional toll can be significant, especially when the final product becomes a painful echo of decline.

### 8.3. A Transformative Alternative

Later, in 2025, Sarah introduced me to a transformative alternative: ElevenLabs^TM^. I was fortunate to access ElevenLabs’ voice banking technology free of charge due to my diagnosis of MND. This Artificial Intelligence (AI)-driven technology was a game-changer in my voice banking journey. Using only a short audio sample from my pre-MND years, ElevenLabs^TM^ generated a synthetic voice that closely resembled my natural voice before disease onset. By simply typing text into a device, selecting my custom voice, and pressing ‘play’, ElevenLabs^TM^ worked their AI algorithm magic, and I could once again hear myself speak—fluently, confidently, and with clarity. I heard not the slow, slurred voice shaped by MND, but the vibrant vocal identity I thought I had lost.

My first encounter with the output from ElevenLabs^TM^ was beyond moving. Although one hopes that they will never have to depend entirely on it, it is ‘vocal insurance’ and an adjunct that gives one some confidence into the future [22]. Unlike earlier systems, ElevenLabs^TM^ lifted my spirits rather than weighing them down.

Using AI-generated speech does come with a learning curve for the user, especially in complex discourses like giving public speeches. Editing for clarity and tone becomes essential. Spelling, phrasing, and abbreviations can all impact how natural the speech sounds. ‘MT’ for ‘mount’, for instance, might be misread. But these are minor technical hurdles compared to the emotional and practical gain of reclaiming your voice.

### 8.4. Speech Pathology Support Is Essential

Throughout this process, the ongoing support of my speech pathologist, Sarah, was not only invaluable, but essential. Navigating voice banking, especially with the added cognitive and emotional load of MND, can be overwhelming. And when you add to that a brand-new IT experience, you could very well give up and walk away. The presence of a knowledgeable clinician helped make a seemingly technical task deeply human and achievable.

### 8.5. Act Early

The process of ElevenLabs^TM^ is straightforward, provided you have an archival voice recording. But even without one, the takeaway was clear: act early. For others facing similar diagnoses, my advice is simple—do not wait. Early recording is crucial in progressive neurological diseases like MND [22,23]. Record your voice while it still feels like your own. Accept that deterioration is an unfortunate part of the journey and prepare for it while you still have the strength and clarity to do so.

Looking back, I regret not starting this journey earlier. Denial, often a companion to degenerative illness, convinced me I did not need to act [8]. I convinced myself that the voice I had would hold steady, even as it was slipping away. My hope is for medical gatekeepers e.g., general practitioners, neurologists, and other medics working with individuals diagnosed with MND and other progressive neurological diseases, to be aware of both current and emerging voice banking technologies. I also hope that they refer early to speech pathology. It is impossible to start voice banking too early; timing is critical [22,23]. I was fortunate to have access to archival recordings from past interviews, but many will not have this advantage. Any delay could mean the difference between preserving a voice you recognise and value, or banking one that already feels compromised. Voice banking is not just a ‘tech’ solution; it is a lifeline for identity, connection, and dignity [22].

### 8.6. Limitations and Ethical Considerations

Although my ElevenLabs^TM^ voice was created from an original Ian Barry recording, I rarely think of it as synthetic. The sound quality is exceptional, with natural flow and clear articulation. While the generated speech does not yet fully capture the emotional nuance of ‘living speech’, it retains enough of the emotional tone from the original recording to convey a striking sense of authenticity. And I understand that this is an area that is actively being developed.

A primary constraint lies in the need to type messages before they can be spoken. The playback quality is so impressive that it is easy to forget the communication still depends on a keyboard, an often-frustrating barrier, especially in spontaneous or emotionally charged conversations. When successfully developed, simultaneous voice conversation devices such as with brain implants will be nothing short of revolutionary [24].

Ethical concerns regarding AI and synthetic voice technologies are valid. Throughout history, humanity has repeatedly demonstrated the capacity to repurpose even the most well-intentioned innovations for harmful ends. Yet we do not withhold beneficial technologies out of fear of potential misuse. The same principle applies here. The transformative potential of voice banking for people living with progressive neurological diseases should not be overshadowed by hypothetical risks. Ethical frameworks and safeguards must of course evolve alongside the technology, but they should not obstruct the life-enhancing benefits this innovation offers to those whose voices, and agency, are being lost [25].

## 9. The Power of My Banked Voice

One of the most powerful applications of my banked voice came when I was invited to deliver an opening statement at a parliamentary inquiry in May 2025 because of a personal drawn-out land acquisition dispute with a government agency. Given the public speaking context, strict time limitations, and the pressure of the setting, I was acutely aware that my MND-affected speech, now slow and effortful, would limit my ability to communicate effectively within the allotted time. A feature of the ElevenLabs^TM^ speech system is fluidity and speed.

I faced a formal and public challenge: delivering an eight-minute statement to the parliamentary inquiry. Aware that my natural speech would be difficult for listeners to follow and would take longer to deliver, I turned to my synthetic voice created through ElevenLabs^TM^. I composed the address with complete creative freedom, as though my speech were unaffected. I no longer had to self-edit to avoid complex multisyllabic words or lengthy sentences. The ability to write without constraint and hear those words spoken fluently in my own voice was profoundly liberating. I typed out my statement and used my synthetic voice to read it. My statement was delivered using my healthy forty-year aged voice before MND’s grasp had taken hold.

When I played the recording for the Minister, I discovered a surprising advantage to using my synthetic voice: the listener was far less likely to interject. The clarity, pacing, and confidence conveyed by the voice gave me space to be heard uninterrupted, and the Minister listened attentively and took notes. This allowed for a more thoughtful and constructive exchange afterward. ElevenLabs^TM^ gave me back not just a tool for communication, but a sense of identity, agency, and voice in the truest sense [22].

## 10. My Voice; My Lifeline

**Figure 3:**
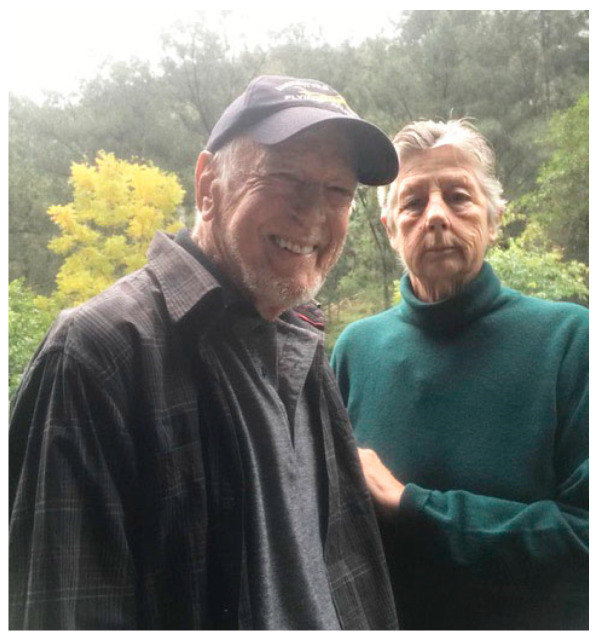
Life with MND: Vicki (my wife and carer) and me at our remote sanctuary (2023).

For my wife Vicki and me, the past several years have brought a relentless succession of challenges. While the pandemic reshaped society, for many living with conditions like MND, Parkinson’s, or multiple sclerosis (MS), the isolation and caution needed to protect one’s immune system were already familiar. In 2020, I retired from a 55-year career, and we retreated to our remote property and embraced a life of seclusion (see Figure 3).

The stress of the land dispute compounded my MND decline, intertwining disease progression with legal and emotional strain. I share this not for sympathy, but to highlight a deeper truth about life with MND—access to communication technology is essential. Tools like ElevenLabs^TM^ have become lifelines. Without them, I would be voiceless in moments that matter most—defending my home, advocating for myself, and maintaining dignity. For many living with progressive neurological diseases, these innovations are not luxuries. They are survival tools.

## 11. Conclusions: A Way Forward

Navigating my own MND journey was at times challenging, frustrating, and disheartening. From early on, I sensed a real disconnect between research and clinical care. It often felt like each part of the system was doing good work, but in isolation. My neurologists were highly skilled and compassionate but often worked in silos with limited interdisciplinary collaboration, reducing opportunities for coordinated and innovative care [26,27]. That sense of fragmentation left me and my family doing much of the coordination ourselves. Compared to other neurological conditions like Parkinson’s or MS, MND often feels like the forgotten cousin—less understood, less resourced, and less prioritised. As a result, this lack of coordinated support leaves many of us, along with our families and carers, feeling isolated and underserved.

If we are to improve outcomes and quality of life for people living with MND, we need more than goodwill. We need systems that communicate, collaborate, and act with urgency. We need targeted and protected funding, early access to innovations like voice banking, and stronger integration between research and care. This can help improve patient outcomes, accelerate research, and enhance the quality of life for individuals with this progressive neurological disease. Just as importantly, we need a community—clinicians, policymakers, researchers, and the public—that recognises the value and dignity of every person living with MND. While the road ahead is complex, I remain hopeful that with greater awareness, coordinated action, and continued technological advances, we can create a more connected and compassionate future for those navigating this disease. Until there is a cure, there should be care.

## 12. A Note from Sarah

Working with Ian has been one of the most profound and humbling experiences of my career. Through his honesty, humour, and fierce determination, he has taught me an immense amount about life, the human spirit, and the role of a speech pathologist; more than any textbook ever could. His insights into living with MND, his courage in the face of loss, and his generosity in sharing his story have left an indelible mark on me—professionally and personally.

### 12.1. Voice Banking: Learning Through Practice and Presence

As a speech pathologist, I have been on a journey of my own, developing the skills and confidence to better support people living with MND. Early in my career, I was nervous about initiating conversations about voice banking. I feared I would not know how to respond if someone became emotional or upset. Over time, however, I developed my skills in incidental counselling, and, more importantly, I learned the value of simply being present. I learnt how important it is to just listen, and leave space for grief, uncertainty, and hope. When I made mistakes or misread a moment, I learned to apologise, pivot, and keep going. My patients, like Ian, have been my greatest teachers.

Supporting people living with MND through the personal and emotional journey of voice banking is both a privilege and a responsibility. Ian was the first person I supported using ElevenLabs’ technology. I remember telling him honestly that I was learning too, and that we would navigate it together. We reached out for help from the company when needed, and together we overcame the challenges. That spirit of collaboration and trust made the process feel empowering, not just for Ian, but also for me as a clinician.

To better support Ian, one of the most valuable ways I learned about the ElevenLabs^TM^ voice banking process was by doing it myself—recording my own voice and following step-by-step videos to guide me through each stage. Experiencing the process firsthand gave me a deeper, more practical understanding of how it works. It also enabled me to demonstrate the result to Ian, which was powerful and reassuring as he considered whether voice banking was right for him.

### 12.2. The Challenge of Late Referrals

One of the biggest challenges I face in my day-to-day work is that many people living with MND are referred too late to speech pathology [28]. The voice of the person with MND has often already changed significantly, and the opportunity to preserve their natural voice has passed. I do explore other options with them, such as using old recordings, voice repair tools, or a voice donor [29,30], but these alternatives can be more expensive, less personal, and may not achieve the same quality or authenticity as a voice banked early in the disease. Furthermore, some individuals may not have any previous recordings of their natural voice.

### 12.3. The Case for Early Voice Banking

Early in the voice banking journey with Ian, I vividly recall him saying, “I wish I had the chance to voice bank earlier. My voice is a big part of who I am. It’s changed too much now. Why aren’t all people living with MND referred to speech pathology right from the start and given information about voice banking?”. His words were honest and heartbreaking—a reminder of what can be lost when opportunities for early intervention are missed. His comment continues to shape both my clinical care and my advocacy for earlier referrals and access to voice banking for everyone with MND. My hope is that our systems will evolve so that voice banking becomes part of standard early care, not a rushed afterthought. This requires the medical gatekeepers to routinely discuss early referrals to speech pathology and present voice banking as an option from the outset.

### 12.4. The Need for Psychological Support

Working with people living with MND has helped me deepen my understanding of the importance of holistic healthcare [31]. Too often, multidisciplinary teams focus on addressing a person’s physical needs, while emotional and psychological support is overlooked [32]. For many people living with MND and their families, conversations about future loss of speech, mobility, and independence can be overwhelming, confronting, and emotional [32]. Access to a psychologist or counsellor from the time of diagnosis is rarely part of standard care. Early psychological support can help people process the journey ahead, navigate uncertainty, and make informed decisions that align with their values [31,33]. I recall Ian saying, “Mentoring and counselling, for me, is part of early action. It is one thing to be given information and to be referred to speech pathology. It is another thing for the sufferer to emotionally and psychologically accept early intervention”. Ian’s words continue to remind me that timely support must include space for emotional readiness. We need to ensure that mental health care is seen as essential, not optional, in MND support [31,33,34].

### 12.5. Mentors and People Living with MND: Our Greatest Teachers

Throughout my journey, I have been deeply grateful for the colleagues who mentored me and generously shared their wisdom, helping me grow both in skills and confidence as I learned to better support people living with MND. Ultimately, it is the people living with MND and their loved ones who have shaped me the most. They have shown me what it means to face life with courage. They have taught me how to live with resilience, love, faith, humour, and creativity. And ultimately, they have shown me how to live fully, even in the face of profound loss and difficulty.

Supporting someone through their MND journey is not just a clinical relationship; it becomes a human connection. In community-based work, clinicians often walk the entire journey with a person, from diagnosis through to end of life, and sometimes even before the diagnosis is confirmed. In those moments, I have witnessed honesty, vulnerability, and strength. I have heard stories of joy, regret, legacy, and meaning. Ian’s voice is a reminder of why this work matters. Thank you to all the people living with MND who continue to teach, challenge, and inspire us to be better listeners, clinicians, and human beings.

### 12.6. A Call to Action

At the heart of this story is a call to action. Voice banking must become a standard part of early MND care, and this requires speech pathologists to keep advocating to medical gatekeepers for timely referrals. People living with MND should also be offered access to psychological support from the beginning to help them process the emotional weight of their diagnosis and make informed choices about their care. As clinicians, we must remain committed to ongoing learning, especially in the face of rapidly evolving technology, so that we can offer the most up-to-date support. And above all, we must be honest, curious, and human: willing to sit in the unknown, to ask questions, and to learn alongside the people we are privileged to support.

## Data Availability

No new data were created or analysed in this study. Data sharing is not applicable to this article.
